# Ultrastructure of antennal sensilla of three fruit borers (Lepidoptera: Crambidae or Tortricidae)

**DOI:** 10.1371/journal.pone.0205604

**Published:** 2018-10-11

**Authors:** Yiping Li, Fangfang Liu, Xiao Du, Zhumei Li, Junxiang Wu

**Affiliations:** 1 Key Laboratory of Plant Protection Resources and Pest Management, Northwest A&F University, Ministryof Education, Yangling, Shaanxi, China; 2 Key Laboratory of Integrated Pest Management on Crops in Northwestern Loess, Shaanxi, China; Nanjing Agricultural University, CHINA

## Abstract

Three fruit borers *Conogethes punctiferalis* (Guenée) (Crambidae), *Grapholita molesta* Busck (Tortricidae), and *Spilonota albicana* Motschulsky (Tortricidae) are serious pests of fruit trees. In this study, their antennal morphology, types of sensilla, and distributions were observed by using SEM (Scanning Electron Microscope). Nine types of sensilla were found on the antennae of *C*. *punctiferalis*, while eight types of sensilla were presented on each of *G*. *molesta* and *S*. *albicana*. The sensilla trichodea with two subtypes were the most abundant sensilla among three fruit borers. Two subtypes of sensillum coeloconica (type I with spines and type II without spines) were observed on the antennae of *C*. *punctiferalis* and *G*. *molesta*. However, sensilla coeloconica (type I) were only found in *S*. *albicana*. Although the sensilla campaniformia were only found on the antennae of *C*. *punctiferalis*, our observations confirm sensilla campaniformia presence in the moths. In addition, the functions of these sensilla were discussed based on previously reported lepidopteran insects. As a result, our study may provide useful information for advanced electrophysiological and behavioral experiments to better understand the mechanisms related to pests control.

## Introduction

*Conogethes punctiferalis* (Guenée)(Crambidae), *Grapholita molesta* Busck (Tortricidae), and *Spilonota albicana* Motschulsky (Tortricidae) are common and serious pests in China [1]. More specifically, the yellow peach moth, *C*. *punctiferalis* is a polyphagous pest, which is widely distributed throughout in China, damaging in buds and fruits of various forests and fruit trees [2]. The oriental fruit moth, *G*. *molesta*, is a worldwide pest that severely damage stone fruit trees, such as pear, peach, plum, apricot, apple, cherry, and other Rosaceae plants [3]. The pear bud moth, *S*. *albicana* is mainly distributed in the east and north of China, attacking hawthorn, apple, pear, peach, and other fruit trees[1]. With the increasing growing cultivated area, the three fruit borers has becoming serious pests of many fruit orchards and crops and cause serious economic loss. At present, the control of them mainly relies on the utility of chemical insecticides. Nevertheless, chemical control also brings with it a series of problems such as insecticide resistance, environmental pollutions and the decrease of biodiversity [4,5]. Consequently, the application of biological controls, including the use of sex pheromones, may become potentially effective measures to suppress the pest [6]. The antennal sensilla of insect is an important organ which can recognize the sex pheromones, so it is the most important step that obtain the ultrastructure of antennal sensilla of this three fruit borers [7].

In insect, the sensillum is a specialized structure of the epidermis, especially occur in the form of hairs, pegs, etc [8,9]. According to the morphology, the sensilla were termed as trichoid, chaetica, coeloconica, basiconca, Böhm bristles, etc. [10]. They play an important role in the feeling of various stimuli (odor, sound, heat, cold, humidity and tactile information) involved in finding suitable habitat and locating mates[11,12,13], on the other hand, play important roles in many behaviors, including detect sex pheromone and host plant volatiles [14,15]. As we know, a lot of studies have characterized the antennal sensilla of various insects, sepecially the structure and function of antennal sensilla in Lepidoptera, have been documented by ultrastructure in many families in the past few decades [16–20]. However, as far as we know, no compared work has been published on the antenna1 sensilla of three fruit borers mentioned above.

In order to better understand their olfactory system related to the biological control of these three fruit borers, we observed and compared the morphology of antennae and type of anternnal sensilla of the male and female between *C*. *punctiferalis*, *G*. *molesta*, *S*. *albicana* adults by using SEM (Scanning Electron Microscope).

## Materials and methods

### Sources of insects

All insects studied were obtained from the orchard of the College of Horticulture of Northwest A&F University, Yangling, Shaanxi Province in China. The fruits (i.e., apple, pear, plum, peach, apricot) which were infested by fruit bores larvae were collected in the orchard, then reared in the insect cages until adults emergence.

### Preparation of specimens

The adults antennae of 10 female and male of each of the three fruit borers were cut under a stereomicroscope by tweezers and sharp blades and washed in 70% ethanol solution (four times, each for 5 s) in an ultrasonic cleaner (KH-250DB; 15°C, 50HZ). After carbon dioxide critical point drying, the antennae were attached to a holder using electric adhesive tape, sputter-coated with gold, examined and photographed with a S-4800 SEM (at 10 kV~15 kV). The antennal sensilla were identified based on their morphology described by Schneider [8] and Na [21].

## Results

### Antennal morphology of *C*. *punctiferalis*

**Fig 1:**
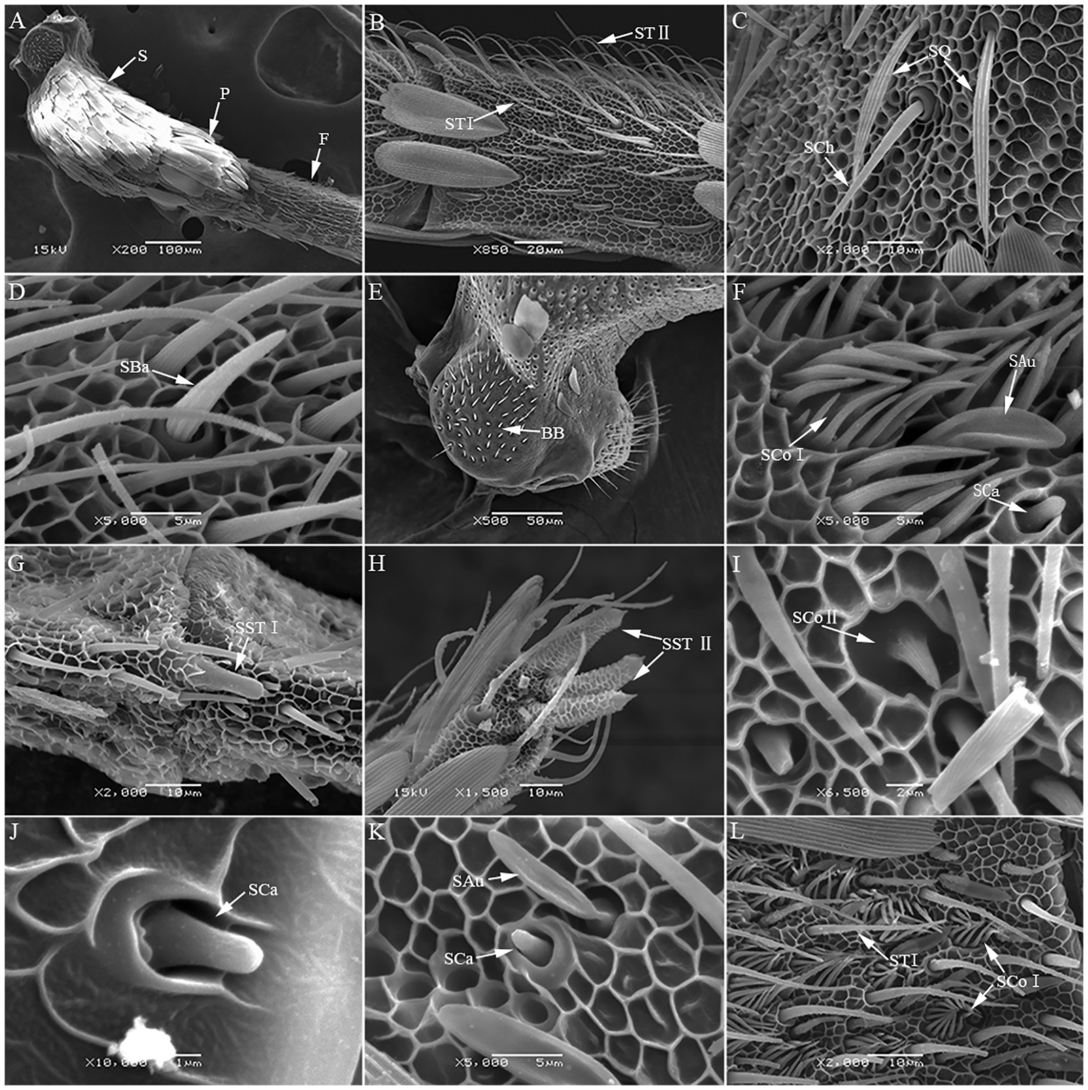
Antennal sensilla of *Conogethes punctiferalis*. A. Morphology of antennae of *Conogethes punctiferalis* S: Scape, P: Pedicel, F: Flagella; B. STI(sensilla trichodea I), STII(sensilla trichodea II); C. SCh(sensilla chaetica), SQ(sensilla squamiformia); D. SBa(sensilla basiconica); E. BB(Böhm bristles); F. SAu(sensilla auricillica), SCoI(sensilla coeloconicaI), SCa(sensilla campaniformia); G. SST I(sensilla styloconica I); H. SSTII(sensilla styloconicaII); I. SCoII(sensilla coeloconicaII); J. SCa(sensilla campaniformia); K. SAu(sensilla auricillica), SCa(sensilla campaniformia); L. ST I(sensilla trichodea I), SCoI(sensilla coeloconica I).

The filamentous antennae of *C*. *punctiferalis* consist of a basal scape, pedicel and elongated flagellum (about 61~75 sub-segments) (Fig 1A). The first two regions are covered with scales, as is on its dorsal surface of the flagellum. In contrast, the ventral surface of the flagellum is equipped with the sensilla. No significant differences are found between sexes, except the number of sub-segment of flagellum (Table 1). Nine types of sensilla are totally found on the antennae of *C*. *punctiferalis*: sensilla trichodea (Type I, II), sensilla chaetica, sensilla basiconica, Böhm bristles, sensilla auricillica, sensilla squamiformia, sensilla styloconica (Type I, II), sensilla coeloconica (Type I, II), and sensilla campaniformia. Notably, sensilla coeloconica can be found on the antennae of female only.

**Table 1:** The differences in antennal sensilla of three fruit borers.

Sensilla types	*Conogethes punctiferalis*		*Grapholitha molesta*		*Spilonota albicana*		
	♀	♂	♀	♂	♀	♂	
sensilla trichodeaI	+	+	+	+	+		+
sensilla trichodeaII	+	+	+	+	+		+
sensilla auricillica)	+	+	+	+	+		+
sensilla chaetica	+	+	+	+	+		+
sensilla basiconica	+	+	+	+	+		+
Böhm bristles	+	+	+	+	+		+
sensilla squamiformia	+	+	+	+	+		+
sensilla styloconicaI	+	+	+	+	+		+
sensilla styloconica II	+	+	+	+	+		+
sensilla coeloconicaI	+	+	+	+	+		+
sensilla coeloconicaII	+	−	+	+	−		−
sensilla coeloconica	+	+	−	−	−		−

Note: “+”indicates “with”; “−”indicates “without”.

Sensilla trichodea (Fig 1B) are the most widely distributed sensilla on the antennae of *C*. *punctiferalis*; they are slender and hair-like, and occur in clusters along the ventral surface of flagellomere. Sensilla trichodea can be divided into two subtypes (Type I and II). Sensilla trichodea Type I (Fig 1L) is straight at the base and taper toward the end. Sensilla trichodea Type II (Fig 1B), is curved at the base, and parallel to the surface of antenna at the terminal end. Sensilla chaetica (Fig 1C), are upright and protrude similar to a thorn with a grooved surface, and they are mainly found on each flagellomere. Sensilla basiconica (Fig 1D) are short and robust with blunt setae. These sensilla present on the distal surface of each sub-segment of flagellum. Böhm bristles (Fig 1E), are thin and sharp with smooth cuticles. They are as in clusters at the base of the scape and pedicel only. Sensilla auricillica (Fig 1F and 1K) are ear-shaped, covered with many small pores on its surface of the cuticular. These sensilla are mainly scattered on the distal of the flagllum. Sensilla squamiformia (Fig 1C) are scale-like and more elongated than scape with a distal end tapering, found along the scape and pedicel among the scales. Sensilla styloconica are thumb-like with a small cone-shape tip. These sensilla are distributed at the distal margin of each sub-segment of flagellum. Sensilla styloconica can also be divided into two subtypes (Type I and II). Sensilla styloconica Type I (Fig 1G) are feeding-bottle shaped with a smooth surface; In contrast, sensilla styloconica Type II (Fig 1H) have a grooved surface with a spine-like tip apically. Sensilla coeloconica (SCo I and SCo II) are only found on the distal of the flagellum. Sensilla coeloconica (SCo I) consist of a submerged central peg surrounded by a ring of cuticular spines, while sensilla coeloconica (SCo II) (Fig 1I) have a central peg only, without a ring of cuticular spines (Fig 1F and 1L). Sensilla campaniformia (Fig 1F, 1J and 1K) are hemispherical-like with a smooth surface, only a few these sensilla are found at the distal margin of sub-segment of flagellum.

### Antennal morphology of *G*. *molesta*

**Fig 2:**
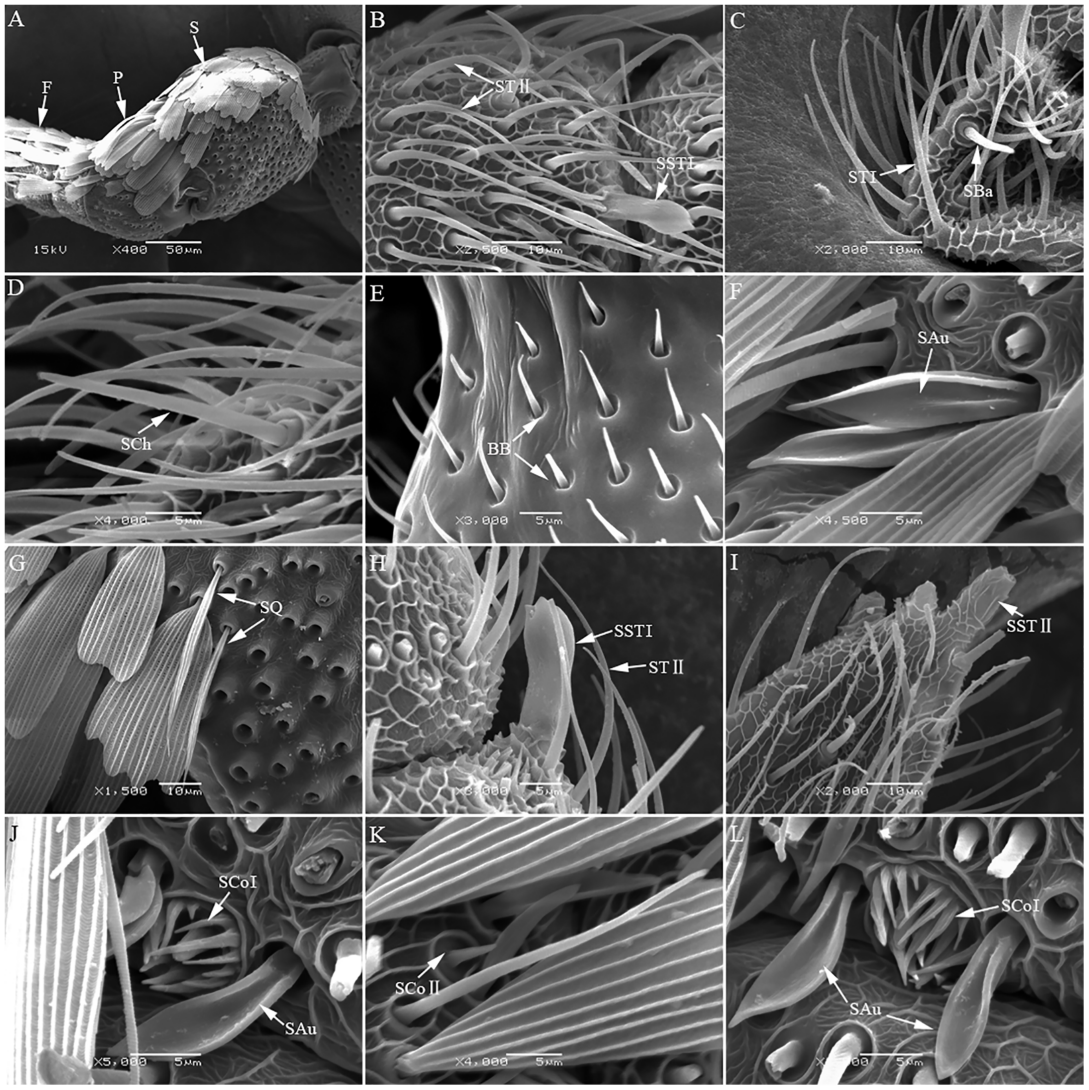
Antennal sensilla of *Grapholitha molesta*. A. Morphology of antennae of *Grapholitha molesta* S: Scape, P: Pedicel, F: Flagella; B. STII(sensilla trichodeaII), SST I(sensilla styloconica I); C. ST I(sensilla trichodea I), SBa(sensilla basiconica); D. SCh(sensilla chaetica); E. BB(Böhm bristles); F. SAu(sensilla auricillica); G. SQ(sensilla squamiformia); H. STII(sensilla trichodeaII), SST I(sensilla styloconica I); I. SSTII(sensilla styloconicaII); J. SCoI(sensilla coeloconicaI), SAu(sensilla auricillica); K. SCoII(sensilla coeloconicaII); L. SCoI(sensilla coeloconicaI), SAu(sensilla auricillica).

The antenna of *G*. *molesta* is filose, and nearly the entire scape and pedicel on the dorsal surface are covered with scales, the flagellum consists of about 41~49 sub-segments (Fig 2A). The number of flagellomere is also different between the sexe*s*. In total, eight types of sensilla were found on the antennae of *G*. *molesta*: sensilla trichodea (ST, Type I and II) (Fig 2C, 2B and 2H), sensilla chaetica (SCh) (Fig 2D), sensilla basiconica (SBa) (Fig 2C), Böhm bristles (BB) (Fig 2E), sensilla auricillica (SAu) (Fig 2F, 2J and 2L), sensilla squamiformia (SQ) (Fig 2G), sensilla styloconica (SST, type I and II) (Fig 2B, 2H and 2I), and sensilla coeloconica (SCo, type I and II) (Fig 2J, 2L and 2K).

### Antennal morphology of *S*. *albicana*

**Fig 3:**
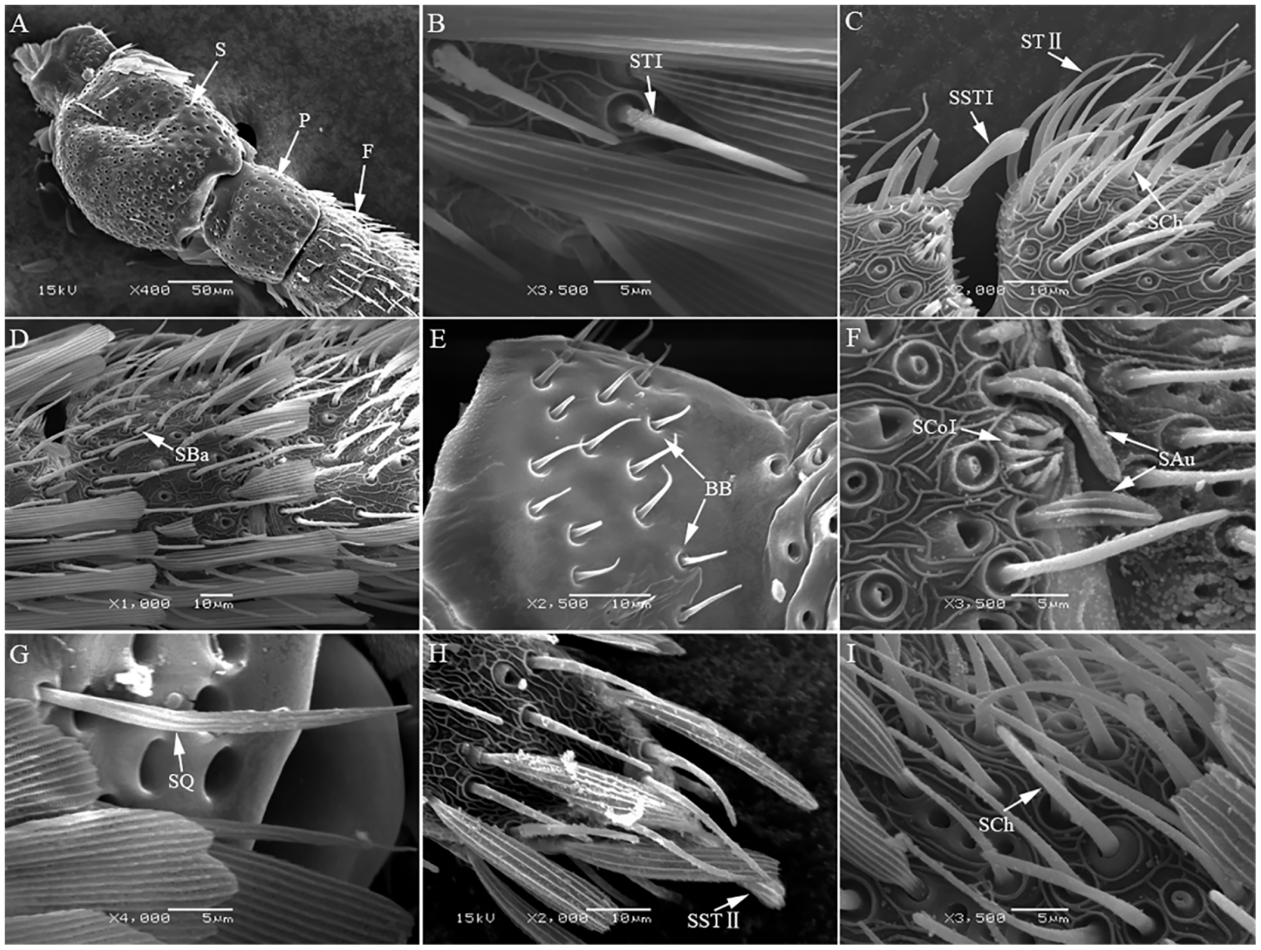
Antennal sensilla of *Spilonota albicana*. A. Morphology of antennae of *Spilonota albicana* S: Scape, P: Pedicel, F: Flagella; B. ST I(sensilla trichodea I); C. STII(sensilla trichodeaII), SST I(sensilla styloconica I), SCh(sensilla chaetica); D. SBa(sensilla basiconica); E. BB(Böhm bristles); F. SAu(sensilla auricillica), SCoI(sensilla coeloconicaI); G. SQ(sensilla squamiformia); H. SSTII(sensilla styloconicaII); I. SCh(sensilla chaetica).

The antennae of *S*. *albicana* is similar to above two species, except the number of flagellum (about 52~56 sub-segments). Similarly, eight types of sensilla are observed totally: sensilla trichodea (Type I, II) (Fig 3B and 3C), sensilla chaetica (Fig 3C and 3I), sensilla basiconica (Fig 3D), Böhm bristles (Fig 3E), sensilla auricillica (Fig 3F), sensilla squamiformia (Fig 3G), sensilla styloconica (Type I, II) (Fig 3C and 3H), and sensilla coeloconica (Fig 3F).

## Discussion

### Sensilla trichodea

Sensilla trichodea, as the most abundant sensilla in this study, were similar in shape between these three fruit borer moths and other lepidopteran insects [22]. According to their size and density, sensilla trichodea could be divided into more subtypes, for example, three subtypes were found on *Ostrinia nubilalis* [23]. But, only two subtypes of sensilla trichodea were identified among the three fruit borer moths in our study. These types of sensilla are olfactory reception of host plant volatiles and sex pheromones [22].

### Sensilla chaetica

Sensilla chaetica have been commonly presented in many lepidopteran insects [24–26]. In this study sensilla chaetica were similar in structure among the three moths. Sensilla chaetica could perceive the movement of antennae as proprio receptors as shown previously [27,28] and considered to be chemoreceptors [29].

### Sensilla basiconica

Sensilla basiconica are larger sensilla with papula surface, sensory cones, and extensively pitted surface [30–33]. Our results showed that sensilla basiconica were morphologically similar in the three moths. This types of sensilla observed in this study also closely resembled to that observed in many other families of Lepidoptera [22]. Sensilla basiconica on the antennae are deduced to possess olfactory function [22,34].

### Böhm bristles

Böhm bristles found in our study morphologically resemble to those observed in many other families of Lepidoptera [35]. In several studies, this type sensilla were considered to be the mechanoreceptors with a proprioceptive function [29,36].

### Sensilla auricillica

Our observation showed that Sensilla auricillica in the three fruit borer moths were consistent with that in butterfly and moth species, even though their distribution and external morphology were variously. Previous studies reported that sensilla auricillica were behaviorally sensitive to plant volatiles in *Cydia pomonella* [35,37].

### Sensilla squamiformia

Sensilla squamiformia were widely distributed among Lepidoptera, though the shape and distribution of these sensilla were different in several reported moth species [22]. However, the function of sensilla squamiformia were seldom reported so far, and these sensilla were inferred to have a mechanoreceptive function [38]. Consequently, further electrophsiological and behavioral studies are necessary to elucidate its function.

### Sensilla styloconica

Two subtype of sensilla styloconica were found on the antennae of the three fruit borer moths in this study, which were identical to those in *Sitotroga cerealella* (Gelechiidae) [39]. The structure and function of these sensilla have already been well studied so far, and they were considered as temperature and humidity receptors [29].

### Sensilla coeloconica

In term of sensilla coeloconica, two subtypes (type I with spines and type II without spines) were observed on the antennae of *C*. *punctiferalis* and *G*. *molesta*, which were similar to those in *Sitotroga cerealella*, and *Manduca sexta* and *Mythimna separate* etc.,[39]. However, sensilla coeloconica (type I) were only found in *S*. *albicana*, which were identical to those observed in skipper butterflies *Parnara* sp. and *Pelopidas* sp. [35]. These sensilla may respond to plant volatiles and also have a temperature and humidity-sensitive function [29].

### Sensilla campaniformia

Generally, a few studies reported that sensilla campaniformia were present in Lepidoptera insects, instead, this type of sensilla were more easily found in Hymenoptera, Hemiptera, Coleoptera and Diptera [30,40–42]. In this study, our observations confirm their presence in the moths, though sensilla campaniformia were only found on the antennae of *C*. *punctiferalis*. Several studies considered sensilla campaniformia as mechanoreceptors while other suggest they were sensitive to temperature and the perception of CO_2_ [22,29].

Previously, Li et al. [43] had already described seven types of antennal sensilla of *C*. *punctiferalis*. However, we confirmed the presence of Böhm bristles and sensilla squamiformia on the antennae of *C*. *punctiferalis*. Meanwhile, Zhang et al. [44] examined the antennae of *G*. *molesta* and found seven types of sensilla. Our results confirmed sensilla squamiformia on the antennae of *G*. *molesta*. For *S*. *albicana*, it is the first time to describe their antennal sensilla in this study, which were similar to those found in *G*. *molesta* and other species from Tortricidae [45,46].

In general, it can be inferred that the antennal sensilla in Lepidoptera are very rich. Correspondingly, sensilla trichodea, sensilla chaetica, sensilla basiconica, sensilla auricillica, sensilla squamiformia, sensilla coeloconica, and sensilla styloconica are commonly present in lepidopteran insects [24–26]. Sensilla chaetica belongs to tactile sensor and mechanical sensor which suggest that this sensor maybe has Location feature [47]. Sensilla basiconica is a kind of chemical sensor and may detect the shock of air [48]. There are many narrow aperture on sensilla auricillica and appears to be accept sound waves [49]. Sensilla coeloconica can be used as feelling the vapour, CO_2_ and plant smell [50,51].

Only their antennal morphology and types of sensilla of these three fruit borers were observed and compared in this study, the size and more information of sensilla were focused in our further studies.

## Conclusions

In summary, the numbers of sub-segmengs of flagellum were varied among these three species, *C*. *punctiferalis* has the highest sub-segmengs of flagellum. Furthermore, we identified nine types of antennal sensilla on *C*. *punctiferalis* and eight types of antennal sensilla on *G*. *molesta*, and *S*. *albicana*. Insects, through evolution and divergence, may evolve different behavioral characteristics [52]. Accordingly, we found the differences on the antennal sensilla of *C*. *punctiferalis*, *G*. *molesta*, and *S*. *albicana*, which may relate to their different hosts and life habits. The comparison between male and female showed that sensilla coeloconicaII existed in *C*. *punctiferalis* and *G*. *molesta* for female, while it existed only in *G*. *molesta* for male. Regard to the sensilla coeloconica, it was found only on the *C*. *punctiferalis* in this study (Table 1). Consequently, our study may provide useful information for taxonomy of Lepidoptera, further advanced electrophysiological and behavioral studies to better understand the mechanisms related to pests control.

## References

[1] Wang JL, Li CL, Yuan KF. The development and control of fruit trees budworm. Mod Agric Sci Tech. 2010: 219–219.

[2] Lu JQ, Wang Z.Y., He K.L., and Liu Y. Research history, progresses and prospects in the yellow peach moth, Conogethes punctiferalis. Acta Phytophy Sin. 2010; 36: 31–38.

[3] Song YQ, Xie XC, Dong JF, Wu JX. cDNA cloning, expression profiling and binding properties of odorant-binding protein GmolOBP3 in the oriental fruit moth, Grapholita molesta (Lepidoptara: Tortricidae). Acta Entomologica Sinica. 2014; 57: 274–285.

[4] Gao XW. Current status and development strategy for chemical control in China. Acta Phytophy Sin. 2010; 36: 19–22.

[5] Lu PF, Huang LQ, Wang CZ. Semiochemicals used in chemical communication in the oriental fruit moth, Grapholitha molesta Busck (Lepidoptera: Tortricidae). Acta Entomologica Sinica. 2010; 53: 1390–1403.

[6] Wang AZ, Li DH, Liang TT, Cai SL. The survey of sex pheromone trapping effects and the occurence for Grapholitha molesta (Busck). Acta Entomologica Sinica. 2012; 21: 203–206.

[7] Wang KQ, Li XM, Liu CL, Liu XL, Wang S, Sun YM. Control of soybean pod borer (Leguminivoraglycinivorella (Mats)) with synthetic sex pheromone. Chin Agric Sci Bull. 2009; 25: 190–193.

[8] Schneider D. Insect Antennae. Annu Rev Entomol. 1964; 9: 103–122.

[9] Altner H, Prillinger L. Ultrastructure of Invertebrate Chemo-, Thermo-, and Hygroreceptors and Its Functional Significance. International Review of Cytology. 1980; 67: 69–139.

[10] Keil TA. Morphology and Development of the Peripheral Olfactory Organs. 1999; 5–47 p.

[11] Altner H, Prillinger L. Ultrastructure of Invertebrate Chemo-, Thermo-, and Hygroreceptors and Its Functional Significance. International Review of Cytology. 1980; 67: 69–139.

[12] Chapman RF (1998) The insects: structure and function: Academic Press 132–141 p.

[13] Godfray HCJ. Parasitoids: behavioral and evolutionary ecology. Environmental Entomology. 1994; volume 24: 483–484(482).

[14] Wang GR, Guo YY, Wu KM. Observation on the Ultrastructures of Antennal Sensilla in Helicoverpa armigera. Sci Agric Sin. 2002; 35: 1479–1482.

[15] Wang H, Wang B, Tian X. Ultrastructural Studies on the Sensilla of Antennae in Ricania sublimbata Jacobi Adult. Acta Entomologica Sinica. 2011; 20: 174–177.

[16] Merivee E, Ploomi A, Rahi M, Bresciani J, Ravn HP, Luik A, et al Antennal sensilla of the ground beetle Bembidion properans Steph. (Coleoptera, Carabidae). Micron. 2002; 33: 429–440. 11976030 10.1016/s0968-4328(02)00003-3

[17] Onagbola EO, Meyer WL, Boina DR, Stelinski LL. Morphological characterization of the antennal sensilla of the Asian citrus psyllid, Diaphorina citri Kuwayama (Hemiptera: Psyllidae), with reference to their probable functions. Micron. 2008; 39: 1184–1191. 10.1016/j.micron.2008.05.002 18573664

[18] Sukontason K, Methanitikorn R, Chaiwong T, Kurahashi H, Vogtsberger RC, Sukontason KL. Sensilla of the antenna and palp of Hydrotaea chalcogaster (Diptera: Muscidae). Micron. 2007; 38: 218–223. 10.1016/j.micron.2006.07.018 16978868

[19] Ahmed T, Zhang TT, Wang ZY, He KL, Bai SX. Morphology and ultrastructure of antennal sensilla of Macrocentrus cingulum Brischke (Hymenoptera: Braconidae) and their probable functions. Micron. 2013; 50: 35–43. 10.1016/j.micron.2013.04.003 23669211

[20] Chang XQ, Shu Z, Liang L, Wang MQ. Insight Into the Ultrastructure of Antennal Sensilla of Mythimna separata (Lepidoptera: Noctuidae). Journal of Insect Science. 2015; 15. 10.1093/jisesa/iev103 PMC4672215 26363060

[21] Na J, Yu WX, Li YP, Dong X, Jiao J. Types and physiological ecology significance of insect antenna sensilla. J Shenyang Normal Univ (Nat Sci). 2008; 26: 213–216.

[22] C FL, K EW, P JR. Sensilla on the antenna and ovipositor of the parasitic wasps trichogramma galloi Zucchi and T. pretiosum Riley (Hym., Trichogrammatidae). Microsc Res Tech. 1999; 45: 313–324. 10.1002/(SICI)1097-0029(19990515/01)45:4/5<313::AID-JEMT15>3.0.CO;2-4 10383124

[23] Hallberg E, Hansson BS, Steinbrecht RA. Morphological characteristics of antennal sensilla in the European cornborer Ostrinia nubilalis (Lepidoptera: Pyralidae). Tissue and Cell. 1994; 26: 489–502. 18621276 10.1016/0040-8166(94)90002-7

[24] Berg BG, Galizia CG, Brandt R, Mustaparta H. Digital Atlases of the Antennal Lobe in Two Species of Tobacco Budworm Moths, the Oriental Helicoverpa assulta (Male) and the American Heliothis virescens (Male and Female). J Comp Neurol. 2002; 446: 123–134. 11932931 10.1002/cne.10180

[25] Burguiere L, Marion-Poll F, Cork A. Electrophysiological responses of female Helicoverpa armigera (Hübner) (Lepidoptera; Noctuidae) to synthetic host odours. J Insect Physiol. 2001; 47: 509–514. 11166315 10.1016/s0022-1910(00)00119-0

[26] Wang X, Jing XU, Liu FY, Chen HB, Jiang-Xing WU, Yong-Jun DU. Ultrastructure of antennal sensilla of Maruca testulalis (Lepidoptera:Pyralidae) adult and its sensory responses to sex pheromone and plant volatiles. Acta Entomologica Sinica. 2008; 51: 1225–1234.

[27] Zhou H, Wu WJ, Niu LM, Fu YG. Antennal sensilla of female Encarsia guadeloupae Viggiani (Hymenoptera: Aphelinidae), a nymphal parasitoid of the spiraling whitefly Aleurodicus dispersus (Hemiptera: Aleyrodidae). Micron. 2013; 44: 365–372. 10.1016/j.micron.2012.09.001 23036370

[28] Romani R, Stacconi MV, Riolo P, Isidoro N. The sensory structures of the antennal flagellum in Hyalesthes obsoletus (Hemiptera: Fulgoromorpha: Cixiidae): a functional reduction? Arthropod Structure & Development. 2009; 38: 473–483. 19682602 10.1016/j.asd.2009.08.002

[29] Ma RY, Du JW. Insect antennal sensilla. Entomol Knowl. 2000; 37: 179–183.

[30] Merivee E, Ploomi A, Rahi M, Luik A, Sammelselg V. Antennal sensilla of the ground beetle Bembidion lampros Hbst (Coleoptera, Carabidae). Acta Zoologica. 2001; 81: 339–350.

[31] Domenichini G. Strutture di Trialeurodes vaporariorum (Westw.) e loro funzioni (Homoptera, Aleyrodidae). Memorie—Societa entomologica italiana. 1982.

[32] Binkmoenen RM. Revision of the African whiteflies (Aleyrodidae), mainly based on a collection from Tchad. 1983; 10: 1–211.

[33] Gerling D. Whiteflies: their bionomics, pest status and management; 1990.

[34] Chinta S, Dickens JC, Baker GT. Morphology and distribution of antennal sensilla of the tarnished plant bug, Lygus lineolaris (Palisot de beauvois) (Hemiptera: Miridae). Int J Insect Morphol Embryol. 1997; 26: 21–26.

[35] Yuan XQ, Gao K, Yuan F, Zhang Y. Ultrastructure of antennal sensilla of four skipper butterflies in Parnara sp. and Pelopidas sp. (Lepidoptera, Hesperiidae). ZooKeys. 2014; 399: 17–27. 10.3897/zookeys.399.7063 PMC4023232 24843250

[36] Li X, Bai S. Ultrastructural studies on the antennal sensilla of Diadegma semiclausum Hellen (Hym., Ichneumonidae). J Henan Agric Univ. 2004; 38: 45–48.

[37] Ansebo L, Ignell R, Lofqvist J, Hansson BS. Responses to sex pheromone and plant odours by olfactory receptor neurons housed in sensilla auricillica of the codling moth, Cydia pomonella (Lepidoptera: Tortricidae). J Insect Physiol. 2005; 51: 1066–1074. 10.1016/j.jinsphys.2005.05.003 15964591

[38] Yu HZ. Research progress of insect Antennal Sensilla. J Anhui Agric Sci. 2007; 35: 4238–4240.

[39] Ma M, Chang MM, Lu Y, Lei CL, Yang FL. Ultrastructure of sensilla of antennae and ovipositor of Sitotroga cerealella (Lepidoptera: Gelechiidae), and location of female sex pheromone gland. Sci Rep. 2017; 7: 40637 28094781 10.1038/srep40637 PMC5240572

[40] Dietz A, Humphreys WJ. Scanning Electron Microscopic Studies of Antennal Receptors of the Worker Honey Bee, including Sensilla Campaniformia. Ann Entomol Soc Am. 1971; 64: 919–925.

[41] Meng Y, Qin D. Fine morphology of the antennae and mouthparts of Dentatissus damnosa (Chou & Lu) (Hemiptera: Issidae). Zool Anz. 2017; 268: 64–74.

[42] Agrawal S, Grimaldi DA, Fox JL. Haltere morphology and campaniform sensilla arrangement across Diptera. Arthropod Struct Dev. 2017; 46: 215–229. 10.1016/j.asd.2017.01.005 28161605

[43] Li Q, Zhang T, Bai S, He K, Wang Q, Li Y, et al Ultrastructure observation and electroantennogram response of Conogethes punctiferalis antennae to corn silk volatiles. Acta Phytophy Sin. 2014; 40: 70–76.

[44] Zhang GH, Song YQ, Tian XL, Wu JX. Ultrastructure of antennal sensilla of oriental fruit moth, Grapholita molesta. J Northwest Sci-Tech Univ Agric For (Nat Sci Ed). 2014; 42: 51–56.

[45] Yang X, Zhao KJ, Wang KQ, Han LL, Yang S. Observation on antennal sensillia of Leguminivora glycinivorellawith scanning electron microscope. Chin J Appl Entomol. 2012; 49: 1321–1326.

[46] Zhao X, Zhang YL, Feng JN. Ultrastructural observation on antennal sensilla of the adult codling moth, Cydia pomonella (L.) (Lepidoptera: Tortricidae). J Northwest Sci-Tech Univ Agric For (Nat Sci Ed). 2012; 40: 119–124.

[47] Cônsoli FL, Kitajima EW, Parra JR. Sensilla on the antenna and ovipositor of the parasitic wasps trichogramma galloi Zucchi and T. pretiosum Riley (Hym., Trichogrammatidae). Microscopy Research & Technique. 1999; 45: 313–324. 10383124 10.1002/(SICI)1097-0029(19990515/01)45:4/5<313::AID-JEMT15>3.0.CO;2-4

[48] Olson DM, Andow DA. Antennal sensilla of female Trichogramma nubilale (Ertle and Davis) (Hymenoptera: Trichogrammatidae) and comparisons with other parasitic Hymenoptera. International Journal of Insect Morphology & Embryology. 1993; 22: 507–520.

[49] VDGVN Wm, Den Otter CJ, Maes FW. Olfactory sensitivity in tsetse flies: a daily rhythm. Chemical Senses. 1998; 23: 351–357. 9669048 10.1093/chemse/23.3.351

[50] Bruce TJ, Cork A. Electrophysiological and behavioral responses of female Helicoverpa armigera to compounds identified in flowers of African marigold, Tagetes erecta. Journal of Chemical Ecology. 2001; 27: 1119–1131. 11504018 10.1023/a:1010359811418

[51] Park KC, Hardie J. Functional specialisation and polyphenism in aphid olfactory sensilla. Journal of Insect Physiology. 2002; 48: 527–535. 12770080 10.1016/s0022-1910(02)00082-3

[52] Hora KH, Roessingh P. Oviposition in Yponomeuta cagnagellus: the importance of contact cues for host plant acceptance. Physiol Entomol. 1999; 24: 109–120.

